# Stage-Specific Rewiring of PI3K–AKT Signaling During Serous Ovarian Cancer Progression: An Integrated Transcriptomic and Network Analysis

**DOI:** 10.3390/ijms27198700

**Published:** 2026-09-29

**Authors:** Iman Jaafar Abdulrahman, Sana Ismael Ameen, Hossein Azizi, Thomas Skutella

**Affiliations:** 1 Obstetrics and Gynecology Department, Akre Obstetrics and Gynecology Hospital, Duhok General Directorate of Health, Ministry of Health, Duhok 42001, Kurdistan Region, Iraq; deimanjaafer@gmail.com; 2 Department of Gynecology and Obstetrics, Faculty of General Medicine, Koya University, Koya 44023, Kurdistan Region, Iraq; sana.ismael@koyauniversity.org; 3 Department of Stem Cells and Cancer, College of Biotechnology, Amol University of Special Modern Technologies, Amol 49767, Iran; 4 Institute for Anatomy and Cell Biology, Medical Faculty, University of Heidelberg, Im Neuenheimer Feld 307, 69120 Heidelberg, Germany; thomas.skutella@uni-heidelberg.de

**Keywords:** cancer progression, GEO analysis, high-grade serous ovarian carcinoma, PI3K–AKT signaling, transcriptomics

## Abstract

High-grade serous ovarian cancer (HGSOC) exhibits substantial molecular heterogeneity during disease progression. We investigated stage-specific changes in PI3K–AKT-associated signaling programs from early tumorigenesis to metastatic disease. Two publicly available microarray datasets, GSE36668 (normal, borderline, and carcinoma; *n* = 4 per group) and GSE73064 (primary tumor, ascites, and metastasis; *n* = 5 per group), were analyzed using differential expression, gene set enrichment, protein–protein interaction network analysis, and hub-gene identification. PI3K–AKT-associated genes were subsequently characterized by functional enrichment, exploratory survival analysis using KM Plotter, and independent stage-stratified validation in TCGA-OV. Survival analyses were considered exploratory because KM Plotter-derived *p*-values were not adjusted for multiple testing. Carcinoma-associated genes were predominantly linked to extracellular matrix remodeling and receptor tyrosine kinase-mediated PI3K signaling. Ascites-associated genes were enriched in integrin-dependent signaling and microenvironmental adaptation, whereas metastasis-associated genes formed inflammatory and survival-related modules involving *IL6*, *JAK1*, *PTK2*, *AREG*, *EIF4E*, and *HSP90B1*. In stage-stratified TCGA-OV analysis, all seven metastasis-associated genes were significantly associated with overall survival in Stage IV disease, supporting the prognostic relevance of the metastatic module. HGSOC progression is characterized by stage-associated reorganization of PI3K–AKT-related signaling rather than uniform pathway activation. The identified molecular programs suggest a transition from RTK-associated proliferative signaling in carcinoma to integrin-dependent survival adaptation in ascites and inflammatory survival signaling during metastasis.

## 1. Introduction

Ovarian cancer remains one of the most lethal gynecological malignancies world-wide, largely due to its biological complexity, asymptomatic progression, and limited early detection strategies [1]. Despite advances in surgical techniques and chemotherapy, survival rates remain dismal, particularly in advanced-stage disease [2]. A deeper understanding of the stepwise molecular changes associated with the transition from normal ovarian epithelium to invasive and metastatic carcinoma is therefore critical for improving patient outcomes.

Serous ovarian carcinomas are broadly classified into low-grade serous carcinoma (LGSC) and HGSOC, which represent biologically distinct disease entities with different molecular characteristics and clinical behavior [3]. LGSC is generally characterized by a more indolent clinical course and frequent alterations in the MAPK pathway, including *KRAS* and *BRAF* [4], and may arise through a continuum involving serous borderline tumors [5]. In contrast, HGSOC is the predominant and more aggressive serous ovarian carcinoma subtype and is characterized by extensive genomic instability, near-universal *TP53* alterations [6], and frequent defects in homologous recombination repair. HGSOC is typically associated with rapid progression, advanced-stage presentation, and widespread peritoneal dissemination [7]. These biological differences indicate that molecular models established for LGSC cannot be directly extrapolated to HGSOC and highlight the need to specifically investigate the molecular programs underlying HGSOC progression [8].

HGSOC accounts for the majority of serous ovarian cancer cases and is frequently diagnosed at an advanced stage, when tumor cells have already disseminated throughout the peritoneal cavity [9]. Its progression involves dynamic changes in tumor-cell proliferation, extracellular matrix remodeling, cell–matrix interactions, inflammatory signaling, and adaptation to the tumor microenvironment [10]. During advanced disease, tumor cells can survive in ascitic fluid [11] and subsequently establish metastatic lesions at distant peritoneal sites [12]. These transitions are accompanied by changes in signaling pathways that regulate cell survival, proliferation, adhesion, migration, and adaptation to cellular stress [13]. Understanding how these molecular programs change across distinct stages of HGSOC progression may therefore provide insights into the biological processes associated with tumor dissemination and metastatic colonization [9].

Among the signaling pathways implicated in ovarian cancer biology, the phosphoinositide 3-kinase (PI3K)/AKT signaling pathway is a central regulator of cell proliferation, survival, metabolism, and adaptation to extracellular signals [14]. Alterations in receptor tyrosine kinase signaling, integrin-mediated interactions, and downstream PI3K–AKT activity may contribute to the acquisition of malignant phenotypes and adaptation to changing microenvironmental conditions [13,15]. However, the extent to which PI3K–AKT-associated molecular programs differ across the stages of HGSOC progression, particularly from early malignant transformation through ascites formation and metastatic disease, remains incompletely characterized [16].

Despite growing molecular insights into HGSOC, stage-associated changes in PI3K–AKT-related signaling programs across early malignant transformation and advanced disease remain incompletely characterized. In this study, we integrated transcriptomic and network-based analyses across distinct stages of HGSOC progression to characterize stage-associated PI3K–AKT-related signaling modules. Stage-specific hub-gene signatures were subsequently evaluated through functional enrichment and exploratory survival analyses and independently examined in stage-stratified TCGA-OV data to assess their cross-cohort reproducibility.

## 2. Results

These two independent GEO datasets were analyzed to identify molecular changes at different stages of disease progression. For the early stage of tumor onset, the GSE36668 dataset was examined, which includes three groups: normal tissue, borderline tumors, and carcinoma samples. To study tumor progression stages, the GSE73064 dataset was analyzed, consisting of three groups: early-stage cancer, ascites, and metastasis (Refer to the Supplementary Files for a detailed analysis of the normalized data).

**Figure 1:**
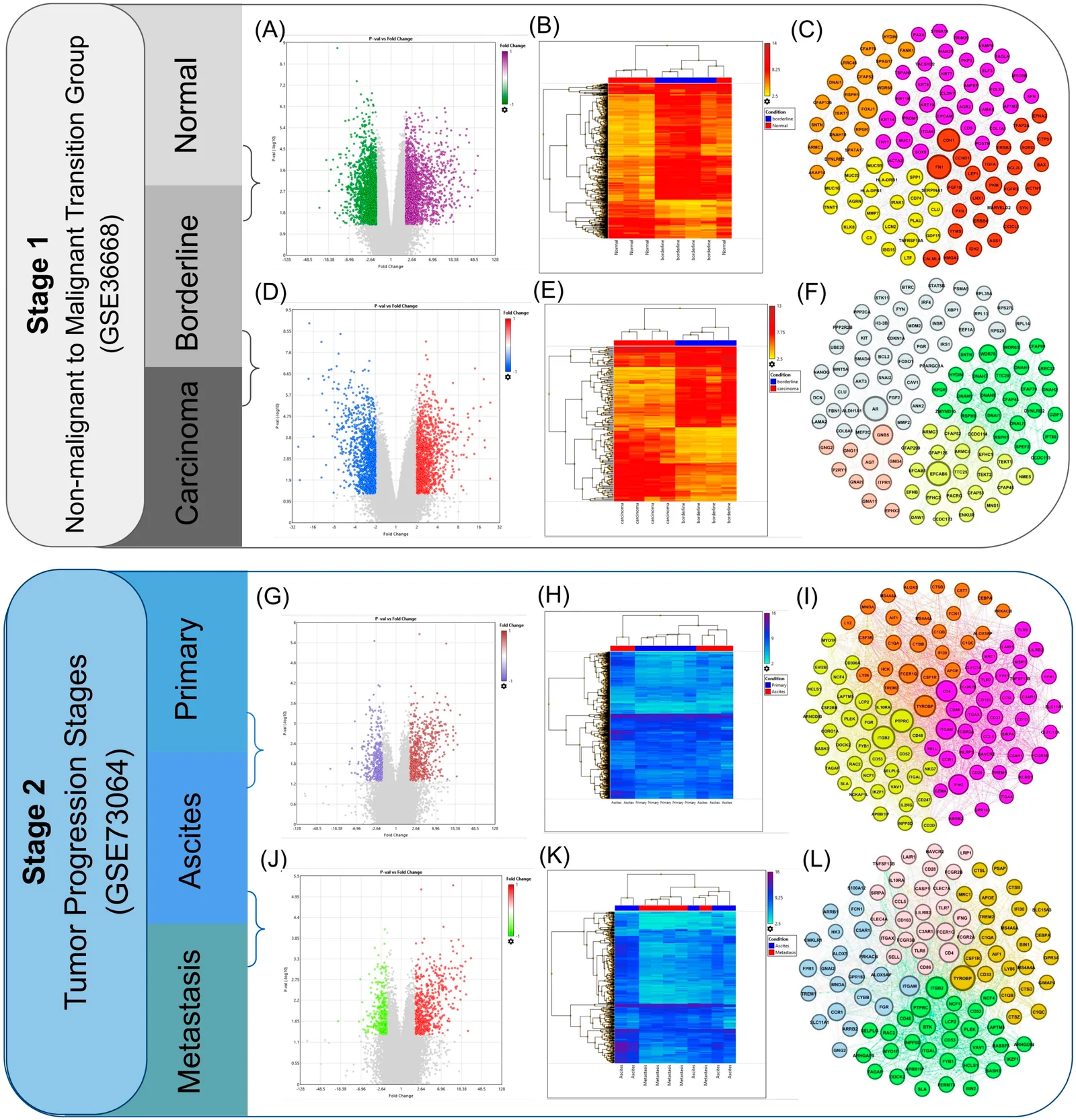
Analysis of non-malignant to malignant transition and tumor progression stages based on gene expression data (GSE36668, GSE73064). (**A**,**D**,**G**,**J**) Volcano plots depicting differential gene expression between groups: normal, borderline, and carcinoma (Stage 1) and primary, ascites, metastasis (Stage 2). (**B**,**E**,**H**,**K**) Heatmaps illustrating hierarchical clustering of gene expression profiles across the specified conditions. (**C**,**F**,**I**,**L**) PPI network visualization showing the clustering patterns of gene expression data for each condition, highlighting key molecular transitions from non-malignant to malignant stages and progression to advanced tumor stages.

Gene expression changes between normal and borderline samples (Figure 1A), carcinoma and borderline (Figure 1D), primary tumor and ascites (Figure 1G), and ascites and metastatic samples (Figure 1J) are shown by volcano plots. Genes with statistically significant expression changes (|log2FC| ≥ 2 and *p*-value < 0.05) are highlighted. Upregulated genes are displayed in red and purple, while downregulated genes are shown in green and blue. The notable asymmetry and the high number of upregulated genes indicate the occurrence of early molecular changes and widespread transcriptomic rearrangements. Hierarchical clustering of genes with differential expression between borderline and normal samples (Figure 1B) and carcinoma and borderline samples (Figure 1E) shows clear separation of the groups. The dendrogram illustrates distinct clustering of carcinoma samples compared to borderline, indicating a significant transcriptomic difference between these two conditions. Despite the clear separation, the intensity of gene expression differences between normal and borderline appears lower compared to carcinoma versus borderline, which aligns with a stepwise progression model from normal to borderline and then to carcinoma. Furthermore, in other comparisons, clustering of the samples also reveals distinct patterns, with samples from the comparisons of primary tumor vs. ascites (Figure 1H) and ascites vs. metastasis (Figure 1K) primarily grouping into separate clusters. Protein–protein interaction network maps constructed from the top 100 upregulated DEGs used for PPI network construction revealed distinct patterns of gene community organization across all comparisons. These networks were generated based on multiple gene topology parameters, including degree, betweenness centrality, closeness centrality, and eigenvector centrality. In the normal versus borderline comparison, four major gene clusters were identified (Figure 1C; Supplementary File S1). Similarly, four distinct gene communities were observed in the borderline versus carcinoma comparison (Figure 1F; Supplementary File S2). The primary versus ascites comparison also demonstrated four clearly separated gene clusters (Figure 1I; Supplementary File S3), whereas three major interconnected gene communities were identified in the ascites versus metastasis comparison (Figure 1L; Supplementary File S4). Overall, each cluster represents groups of highly interconnected genes whose coordinated activity may contribute to the enrichment of distinct biological functions during ovarian cancer progression.

**Figure 2:**
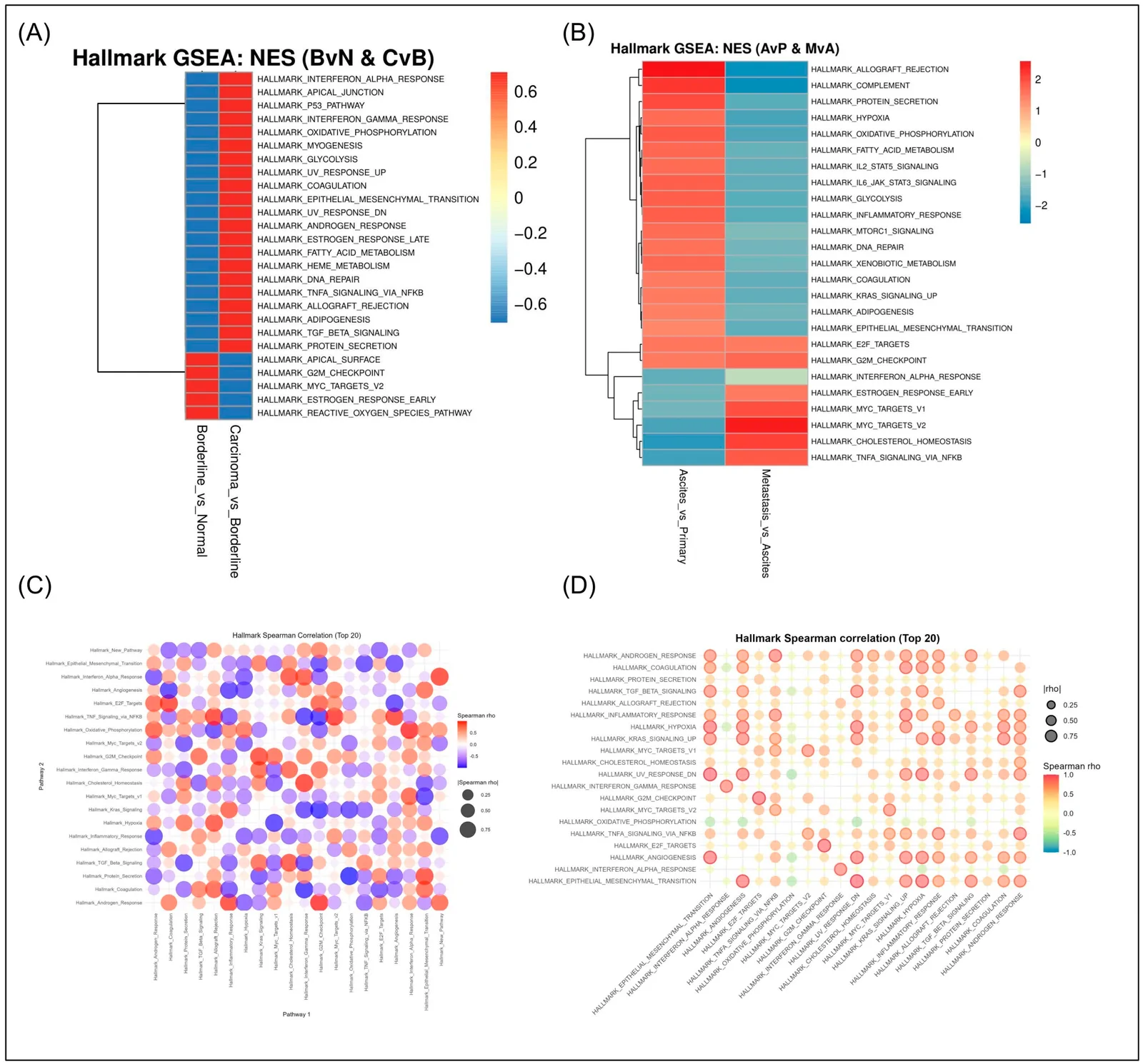
Hallmark gene set enrichment analysis and ssGSEA-derived pathway activity correlation analysis. (**A**) Heatmap showing NES obtained from Hallmark GSEA for comparisons of borderline versus normal (BvN) and carcinoma versus borderline (CvB) samples. (**B**) Heatmap showing NES values from Hallmark GSEA for comparisons of ascites versus primary (AvP) and metastasis versus ascites (MvA) samples. (**C**) Spearman correlation analysis of Hallmark gene sets based on ssGSEA-derived pathway activity scores in stage 1 (non-malignant to malignant transition). (**D**) Spearman correlation analysis of Hallmark gene sets based on ssGSEA-derived pathway activity scores in stage 2 (tumor progression). For visualization, the 20 gene sets showing the strongest correlation patterns are displayed in panels (**C**,**D**). The size of the circles represents the absolute value of the Spearman correlation coefficient (|ρ|), whereas the color indicates the direction of the correlation, with red representing positive and blue representing negative correlations.

This chart represents the results of Gene Set Enrichment Analysis (GSEA), showing the normalized enrichment scores (NES) of Hallmark pathways across normal, borderline, and carcinoma samples. The data are compared using NES, indicating which biological pathways are more active in each group (Figure 2A). The results of the GSEA show that in carcinoma cells, pathways related to inflammatory responses (such as Interferon Alpha Response and TNF-α Signaling via NFKB) are significantly more active compared to normal and borderline cells. These changes are particularly relevant to immune responses and chronic inflammation in cancer and can help explain the role of inflammation in tumorigenesis and cancer progression. On the other hand, DNA repair-related pathways are less active in carcinoma cells, which may indicate structural and functional defects in genomic repair and cell proliferation processes. In borderline cells, inflammatory response pathways and some metabolic pathways are activated, indicating that these cells are progressing toward tumor-like characteristics but have not yet reached the full carcinoma stage. Furthermore, Spearman correlation analysis highlights the relationships between key cancer-related pathways, such as those involved in inflammation, oxidative phosphorylation, and DNA repair (Figure 2C). The analysis reveals significant correlations between the pathways, especially between inflammatory response pathways (e.g., TNF-α signaling) and cellular processes like oxidative stress and metabolic shifts. These findings indicate coordinated changes in inflammation-, metabolism-, and DNA repair-related pathway activity across the analyzed samples and may help characterize transcriptional changes associated with tumor progression.

The results of GSEA are presented, comparing Ascites vs. Primary and Metastasis vs. Ascites in high-grade serous ovarian cancer (Figure 2B). The data, expressed as NES, provide a detailed view of pathway activity across primary, ascites, and metastatic HGSOC samples. The analysis shows that certain pathways, particularly those related to immune responses (e.g., TNFα Signaling via NFKB), metabolism, and inflammation (e.g., Inflammatory Response, Oxidative Phosphorylation, Fatty Acid Metabolism), are significantly activated in Ascites and Metastasis compared to Primary cancer cells. These results suggest that as cancer progresses from Primary to Ascites and eventually to Metastasis, there is a notable shift towards enhanced inflammatory and metabolic activity, potentially contributing to the survival and expansion of cancer cells in hostile microenvironments. The Hallmark of EMT pathway is also more active in the Ascites and Metastasis stages, consistent with increased activation of EMT-associated transcriptional programs that is characteristic of metastatic cancer. This finding highlights the critical role of EMT in facilitating the migration of tumor cells from primary tumors to distant organs. The Spearman correlation analysis represents the top 20 Hallmark gene sets (Figure 2D). Notably, several pathways such as Hallmark_Epithelial_Mesenchymal_Transition and Hallmark_Interferon_Alpha_Response exhibit strong positive correlations with other biological processes. This suggests that these pathways may be functionally interconnected, particularly in the context of EMT and immune responses during cancer progression. Additionally, several gene sets, including Hallmark_Angiogenesis, Hallmark_TNF_Signaling_via_NFKB, and Hallmark_Interferon_Gamma_Response, showed positive correlations in their single-sample GSEA (ssGSEA) scores, indicating coordinated variation in pathway activity across the analyzed samples. These correlated pathway activity patterns may reflect shared biological changes involving inflammation, immune-related processes, and angiogenesis during disease progression.

**Figure 3:**
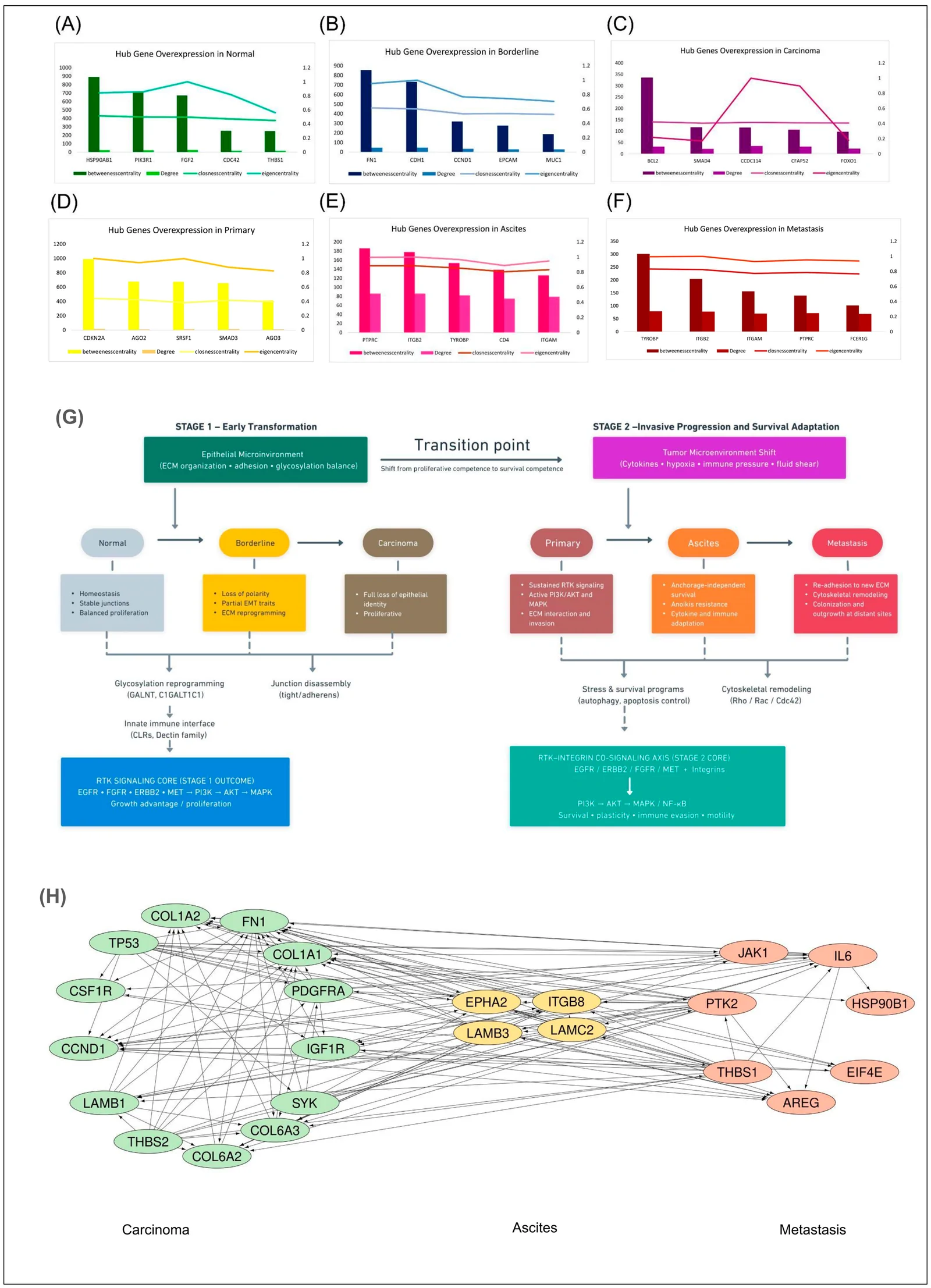
Hub-Gene Analysis and Pathway Transition in Cancer Stages. (**A**–**F**) Centrality-based hub genes across Normal, Borderline, Carcinoma, Primary, Ascites, and Metastasis stages, selected as the five genes with highest composite rank across degree, betweenness, closeness, and eigenvector centrality within each comparison’s top-100 upregulated DEG network. Note that this unfiltered, centrality-only selection is susceptible to interactome-topology artefacts: the Ascites and Metastasis hub genes (*PTPRC*, *ITGB2*, *TYROBP*, *CD4*, *ITGAM*, *FCER1G*) constitute a pan-leukocyte/myeloid panel and should be interpreted as a mixed tumor-immune signal rather than a tumor-cell-intrinsic signature (see Supplementary Files S6A and S6B); similarly, the motile-cilia genes *CCDC114* and *CFAP52* in panel (**C**) reflect the dense cilia/axoneme module in the STRING interactome. (**G**) Schematic of the Reactome-derived shift from Stage 1 (Early Transformation) to Stage 2 (Invasive Progression and Survival Adaptation); components in panel G not directly identified as DEGs in this dataset (e.g., glycosylation- and innate immune-related genes) are included from established literature for context. (**H**) STRING/Cytoscape PPI network of PI3K–AKT pathway-associated genes, color-coded by stage (green: Carcinoma; orange: Ascites; red: Metastasis). The genes in panel (**H**) were selected by pathway membership, not by network centrality, and are therefore a distinct, non-overlapping selection from those in panels (**A**–**F**); none of the leukocyte markers noted above are members of the PI3K–AKT pathway gene sets and none appear in panel (**H**). Arrows indicate the direction of the proposed molecular transitions or signaling relationships between connected components; they represent established or inferred relationships based on the underlying pathway and network analyses.

Network analysis identified stage-specific hub genes based on their network-topology profiles. The five highest-ranked hub genes for each stage are summarized in Table 1, while their corresponding network profiles are visualized in Figure 3A–F. In the Normal group (Figure 3A), *HSP90AB1*, *PIK3R1*, *FGF2*, *CDC42*, and *THBS1* showed prominent network-centrality values. In the Borderline group (Figure 3B), *FN1*, *CDH1*, *CCND1*, *EPCAM*, and *MUC1* were prioritized as hub genes. In the Carcinoma group (Figure 3C), *BCL2, SMAD4*, *CCDC114*, *CFAP52*, and *FOXO1* showed the highest composite network rankings. In the Primary tumor group (Figure 3D), *CDKN2A*, *AGO2*, *SRSF1*, *SMAD3*, and *AGO3* were identified as hub genes.

Also, in the ascites samples (Figure 3E), the genes *PTPRC*, *ITGB2*, *TYROBP*, *CD4*, and *ITGAM* showed the highest composite centrality, and in the metastasis stage (Figure 3F), *TYROBP*, *ITGB2*, *ITGAM*, *PTPRC*, and *FCER1G* were identified. These constitute a pan-leukocyte and myeloid marker panel (*PTPRC* = *CD45*, *TYROBP* = *DAP12*, *ITGAM* = *CD11b*) rather than a tumor-cell-intrinsic signature. Because GSE73064 specimens were EpCAM-sorted for epithelial enrichment, we examined leukocyte marker expression directly: absolute expression was detectable but did not differ significantly between groups (Kruskal–Wallis *p* = 0.47 for *PTPRC*, *p* = 0.21–0.59 for *TYROBP*, *ITGAM*, *FCER1G*, and *CD4*; Supplementary Files S6A and S6B). Their prominence in the centrality ranking therefore most plausibly reflects the high intrinsic connectivity of immune-gene modules within protein–protein interaction databases rather than a genuine stage-specific difference in immune content. We accordingly present these unfiltered hub genes descriptively and do not interpret them as tumor-cell-intrinsic drivers; the manuscript’s mechanistic conclusions rest on the pathway-filtered gene sets described below, which contain none of these markers.

To provide an integrated interpretation of the enriched molecular pathways derived from upregulated genes at each stage of tumor progression, Reactome pathway analysis results were summarized in a two-stage schematic model (Figure 3G). This model illustrates the gradual transition from normal tissue to invasive malignancy based on the dominant functional programs characterizing each stage.

In Stage 1 (Early Transformation), encompassing comparisons among normal, borderline, and carcinoma samples, enriched pathways were predominantly associated with the epithelial microenvironment, including cell–cell adhesion, ECM organization, and glycosylation balance. Normal cells were characterized by preserved homeostasis, stable junctions, and balanced proliferation. In contrast, borderline lesions exhibited loss of cellular polarity, partial EMT, and ECM reprogramming, reflecting an intermediate and phenotypically unstable state. Progression to carcinoma was marked by a complete loss of epithelial identity and increased proliferative capacity. At this stage, enrichment of receptor tyrosine kinase (RTK)–related signaling, including *EGFR*, *ERBB2*, *FGFR*, and *MET*, together with downstream PI3K–AKT–MAPK pathways, was primarily interpreted as conferring a proliferative and growth advantage.

The transition point in this model reflects a functional shift from proliferative competence toward survival competence, indicating that the transcriptional profiles increasingly reflected survival-related and microenvironment-associated signaling relative to growth-related programs. In Stage 2 (Invasive Progression and Survival Adaptation), which includes comparisons among primary tumors, ascites, and metastatic lesions, enriched pathways were largely linked to tumor microenvironmental remodeling, including hypoxia, inflammatory and immune signaling, cytokine exposure, and mechanical stress. Primary tumors exhibited sustained RTK activation together with active PI3K/AKT and MAPK signaling, supporting ECM interaction and invasive behavior. Ascites-derived tumor cells were characterized by anchorage-independent survival, anoikis resistance, and adaptation to immune and cytokine stress, consistent with the activation of stress and survival programs such as autophagy and apoptosis regulation. Metastatic cells displayed enhanced re-adhesion to a new ECM, cytoskeletal remodeling, and activation of colonization and outgrowth programs at distant sites.

At this stage, the RTK–integrin co-signaling axis emerged as a central functional core, integrating signals through PI3K–AKT, MAPK, and NF-κB pathways to promote cell survival, phenotypic plasticity, immune evasion, and increased motility. Overall, this schematic model indicates that tumor progression from early transformation to metastasis is accompanied by changes in growth signaling, survival pathways, microenvironmental interactions, and adaptive cellular states.

Although the Carcinoma group originated from a distinct dataset (GSE36668) representing early-stage tumorigenesis, while Ascites and Metastasis were derived from GSE73064 representing advanced progression, these stages were intentionally analyzed within a single integrated network to model a continuous biological trajectory rather than a directly matched statistical comparison. Carcinoma represents the convergence point of Stage 1 (early transformation), while Ascites and Metastasis represent sequential steps of Stage 2 (invasive progression); combining their PI3K–AKT-associated hub genes therefore enables visualization of how proliferative signaling established in early carcinoma is functionally inherited and reprogrammed toward survival and dissemination programs in later stages, consistent with the two-stage progression model proposed in Figure 3G. To resolve the gene-level architecture of the PI3K–AKT axis identified as the central signaling core across disease stages, hub genes from the Carcinoma, Ascites, and Metastasis groups were filtered against the PI3K–AKT pathway gene set and visualized as an integrated PPI network (Figure 3H). The network showed clear stage-dependent clustering: Carcinoma genes (*FN1*, *COL1A1*, *COL1A2*, *TP53*, *PDGFRA*, *CSF1R*, *IGF1R*, *CCND1*, *LAMB1*, *SYK*, *THBS2*, *COL6A2*, *COL6A3*) formed the most densely connected community, reflecting ECM remodeling and RTK signaling; Ascites genes (*LAMC2*, *EPHA2*, *ITGB8*, *LAMB3*) clustered around integrin-mediated adhesion; and Metastasis genes (*IL6*, *JAK1*, *AREG*, *THBS1*, *EIF4E*, *PTK2*, *HSP90B1*) formed a cytokine/growth-factor-driven community.

**Figure 4:**
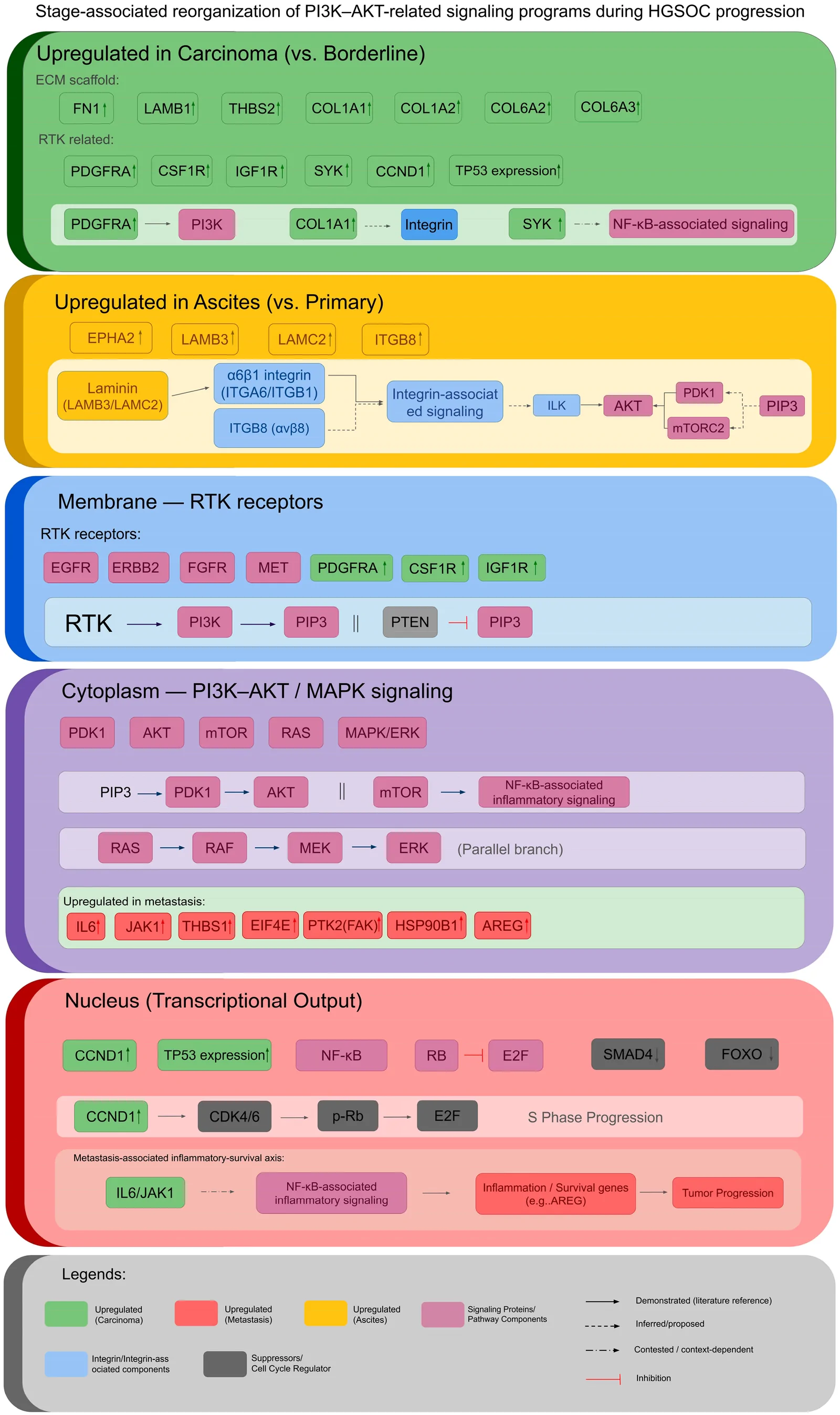
Stage-associated reorganization of PI3K–AKT-related signaling programs during HGSOC progression. Differentially expressed genes identified in carcinoma, ascites, and metastatic samples are mapped onto key signaling modules, including extracellular matrix remodeling, RTK activation, integrin signaling, PI3K–AKT and MAPK pathways, and nuclear transcriptional regulators. In the carcinoma-associated module, RTK-related signaling and its connection with PI3K are supported by established literature [17]. In the ascites-associated module, integrin- and focal-adhesion-related signaling is supported by previous studies [18]. The canonical PI3K–AKT relationships are presented as established signaling relationships and are contextualized by previous studies of PI3K–AKT signaling in ovarian cancer [14,15,16]. The schematic illustrates the coordinated signaling modules associated with proliferation, survival, inflammation, angiogenesis, and metastatic progression. Upward arrows (↑) indicate increased expression of the corresponding genes in the indicated disease stage. Relationships represented by dashed or context-dependent arrows are intended as inferred or proposed interactions rather than direct experimental evidence. Upward arrows (↑) indicate increased expression of the corresponding genes in the indicated disease stage.

To further resolve the stage-specific organization of the PI3K–AKT signaling axis, PI3K–AKT-associated hub genes identified in the carcinoma, ascites, and metastasis groups were integrated into a layered schematic model spanning extracellular matrix remodeling, membrane receptors, intracellular kinase signaling, and nuclear transcriptional outputs (Figure 4). This integrative representation revealed a progressive reorganization of signaling modules rather than activation of a single linear pathway. In the carcinoma stage, the predominant molecular alterations involved extracellular matrix remodeling and receptor tyrosine kinase (RTK)-associated signaling. ECM components including *FN1*, *LAMB1*, *THBS2*, *COL1A1*, *COL1A2*, *COL6A2*, and *COL6A3*, together with RTK-related genes (*PDGFRA, CSF1R*, and *IGF1R*) formed the principal upstream signaling module. These alterations converged on PI3K activation through RTK and integrin-associated signaling, indicating that early carcinoma was characterized by proliferative-associated transcriptional programs involving ECM remodeling and growth-factor responsiveness. In contrast, ascites-associated hub genes defined a transitional signaling state characterized by enhanced integrin-mediated signaling. Upregulation of *EPHA2*, *ITGB8*, *LAMB3*, and *LAMC2* highlighted activation of the α5β1/α1β1–ITGB8–ILK–PDK1/mTORC2–AKT signaling axis, suggesting adaptation to anchorage-independent survival and resistance to microenvironmental stress during peritoneal dissemination. Metastatic progression was characterized by enrichment of inflammatory and survival-associated signaling downstream of PI3K–AKT. Metastasis-specific hub genes including *IL6*, *JAK1*, *THBS1*, *EIF4E*, *PTK2 (FAK)*, *HSP90B1*, and *AREG* were integrated within AKT-, MAPK-, and NF-κB-associated signaling modules, indicating reinforcement of inflammatory signaling, translational activation, focal adhesion remodeling, and survival pathways associated with metastatic colonization. At the nuclear level, carcinoma-associated activation of *CCND1* and *TP53* expression was linked to cell-cycle progression through the CDK4/6–RB–E2F axis, whereas metastatic signaling preferentially converged on NF-κB-mediated inflammatory and survival programs. Collectively, the schematic demonstrates that ovarian cancer progression is accompanied by a gradual shift from RTK-driven proliferative signaling in carcinoma toward integrin-dependent survival adaptation in ascites and inflammatory survival signaling during metastasis.

To functionally validate the gene clusters represented in the integrated PI3K–AKT schematic, hub genes from the Carcinoma, Ascites, and Metastasis groups were independently submitted to Enrichr for Gene Ontology enrichment analysis (Supplementary Table S1). Carcinoma-associated genes were enriched in kinase-driven motility programs (e.g., peptidyl-tyrosine phosphorylation, positive regulation of cell migration); ascites-associated genes in cell motility- and angiogenesis-associated processes (e.g., positive regulation of cell motility, regulation of angiogenesis); and metastasis-associated genes predominantly in cytokine signaling and cell adhesion regulation (e.g., interleukin-6-mediated signaling, negative regulation of integrin-mediated cell adhesion). These enrichment patterns corroborate the functional roles assigned to each stage within the schematic model in Figure 4.

**Figure 5:**
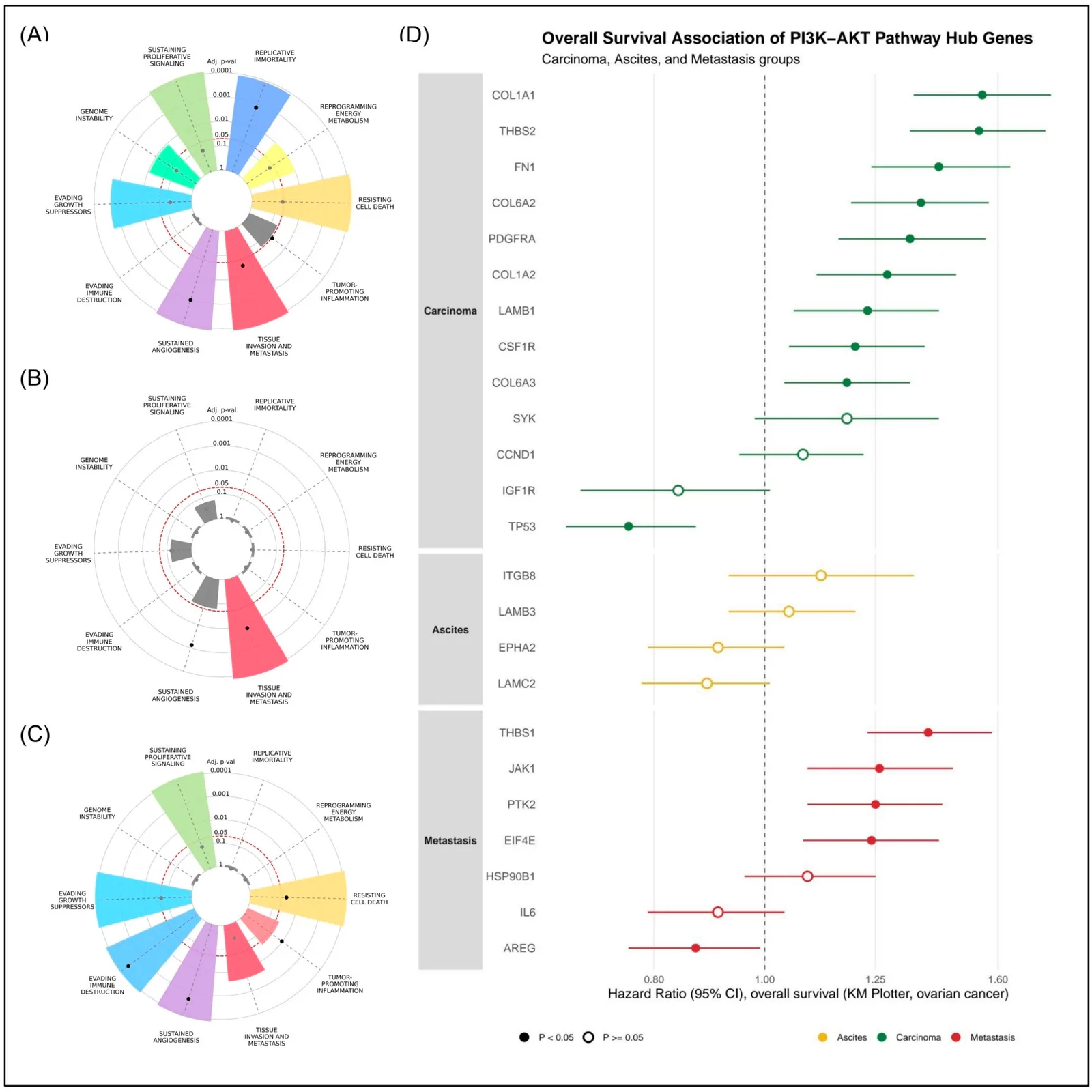
Hallmark enrichment and survival relevance of PI3K–AKT-associated hub genes. (**A**–**C**) Polar bar plots of hallmark enrichment (Core cancer hallmark gene set, *n* = 1574) for upregulated hub genes in Carcinoma (**A**), Ascites (**B**), and Metastasis (**C**); bar length reflects adjusted *p*-value (scale 0.0001–1), red dashed circle marks *p* = 0.05. (**D**) Forest plot of overall-survival hazard ratios (HR, 95% CI) across the three groups, compiled from individual KM Plotter analyses (Supplementary Figure S1) in R (ggplot2); filled circles: *p* < 0.05, open circles: *p* ≥ 0.05. The vertical dashed line indicates the null value (HR = 1.0).

To contextualize stage-specific hub genes within broader oncogenic programs, upregulated genes from each group were mapped onto the ten hallmarks of cancer using the Core cancer hallmark gene set. Carcinoma-associated hub genes showed significant enrichment (adjusted *p* < 0.05) across eight of the ten hallmarks—Sustaining Proliferative Signaling, Replicative Immortality, Genome Instability, Evading Growth Suppressors, Evading Immune Destruction, Sustained Angiogenesis, Tissue Invasion and Metastasis, and Resisting Cell Death—with only Reprogramming Energy Metabolism and Tumor-Promoting Inflammation failing to reach significance, consistent with the functional characteristics of the PI3K–AKT-associated network modules identified in this study (Figure 5A). Ascites-associated hub genes showed markedly more restricted enrichment, with only Tissue Invasion and Metastasis reaching significance, reflecting a transitional functional signature centered on integrin-mediated adhesion and consistent with the smaller hub-gene set available for this group (*n* = 4) (Figure 5B). Metastasis-associated hub genes displayed significant enrichment across Sustaining Proliferative Signaling, Resisting Cell Death, Evading Growth Suppressors, Evading Immune Destruction, Sustained Angiogenesis, and Tissue Invasion and Metastasis, consistent with the survival- and inflammation-associated functional features represented within the metastasis-associated network module (Figure 5C). To further determine whether stage-specific PI3K–AKT hub genes carry prognostic relevance, overall survival analysis was performed for each gene using the KM Plotter database (Figure 5D; Supplementary Figure S1. Among Carcinoma-associated hub genes, ECM- and RTK-related genes including *COL1A1* (HR = 1.55, *p* = 1.8 × 10^−10^), *THBS2* (HR = 1.54, *p* = 4.3 × 10^−10^), *FN1* (HR = 1.42, *p* = 7.3 × 10^−7^), *COL6A2* (HR = 1.37, *p* = 6.3 × 10^−6^), *PDGFRA* (HR = 1.34, *p* = 9.5 × 10^−5^), *COL1A2* (HR = 1.28, *p* = 5.1 × 10^−4^), *LAMB1* (HR = 1.23, *p* = 0.0048), *CSF1R* (HR = 1.20, *p* = 0.0074), and *COL6A3* (HR = 1.18, *p* = 0.011) were significantly associated with poorer overall survival, whereas *TP53* (HR = 0.76, *p* = 5.3 × 10^−5^) was associated with improved survival. In contrast, none of the Ascites-associated hub genes (*ITGB8*, *LAMB3*, *EPHA2*, and *LAMC2*) reached statistical significance, consistent with their proposed role as transitional, microenvironment-adaptive markers rather than intrinsic drivers of aggressive disease. Among Metastasis-associated hub genes, *THBS1* (HR = 1.39, *p* = 2.8 × 10^−7^), *JAK1* (HR = 1.26, *p* = 0.0014), *PTK2* (HR = 1.25, *p* = 0.0011), and *EIF4E* (HR = 1.24, *p* = 0.0029) were significantly associated with worse survival, while *AREG* (HR = 0.87, *p* = 0.03) showed a modest protective association. Collectively, these findings indicate that hub genes identified through network analysis of early carcinoma and metastatic lesions carry independent prognostic value in an external ovarian cancer cohort, whereas ascites-associated hub genes largely reflect a transitional adaptive state without strong survival impact.

To address the stage-heterogeneity of the KM Plotter cohort, hub genes were independently reevaluated in TCGA-OV using stage-matched sample stratification (Carcinoma: Stage I–III, *n* = 358; Ascites and Metastasis: Stage IV) (Supplementary Figure S2). Validation strengthened substantially for the Metastasis-associated hub genes: all seven reached significance in TCGA Stage IV (*p* range 7.5 × 10^−3^ to 1.6 × 10^−8^), compared with five of seven in KM Plotter, with five of seven genes (*EIF4E*, *HSP90B1*, *JAK1*, *PTK2*, *THBS1*) showing concordant risk direction across both platforms. Notably, *AREG* and *IL6* diverged in direction between cohorts: *AREG* was a significant protective factor in KM Plotter (HR = 0.87, *p* = 0.03) but a strongly significant risk factor in TCGA Stage IV (HR = 1.25, *p* = 1.6 × 10^−8^), and *IL6* shifted from non-significant in KM Plotter (HR = 0.91, *p* = 0.16) to a significant risk factor in TCGA Stage IV (HR = 1.35, *p* = 1.0 × 10^−7^), suggesting that inflammatory/cytokine signaling captured by these genes is particularly relevant to genuinely advanced, metastatic disease and may be diluted or obscured in stage-heterogeneous cohorts. The Ascites-associated hub genes showed directional concordance for three of four genes (75%; *EPHA2*, *ITGB8*, *LAMC2*), with *LAMB3* discordant though non-significant in both cohorts, independently supporting their proposed role as transitional, microenvironment-adaptive markers rather than intrinsic prognostic drivers (Supplementary Table S2).

For the Carcinoma-associated hub genes, initial median-split analysis in TCGA showed minimal concordance with KM Plotter (1/13 genes, 7.7%), with most genes trending toward a protective association in TCGA versus a risk association in KM Plotter. Because KM Plotter uses an optimal (best) cutoff-selection strategy rather than a median split, a sensitivity analysis using a matched best-cutoff approach was performed (Supplementary Figure S4); this improved directional concordance to 9/13 genes (69.2%), indicating that cutoff-selection method, rather than true biological reversal, accounted for most of the initial discordance. Four genes remained discordant regardless of cutoff method (*FN1*, *SYK*, *IGF1R*, *TP53*); the *TP53* discordance is consistent with the near-universal *TP53* mutation status of HGSOC, which decouples transcript-level expression from protein function (see Limitations). Across all 24 hub genes, and using the matched-cutoff Carcinoma estimates, overall cross-cohort directional concordance was 17/24 (71%) (Supplementary Figure S3 and Table S2).

## 3. Discussion

This study characterizes stage-specific molecular programs associated with HGSOC progression from early tumorigenesis to advanced metastatic stages. Inflammatory signaling, immune modulation, and metabolic reprogramming emerged as central processes shaping the tumor microenvironment across this trajectory.

Enrichment of inflammatory pathways such as TNF-α signaling via NF-κB and interferon-alpha response in carcinoma and metastatic stages was associated with transcriptional programs related to tumor growth and progression. This is consistent with prior work identifying inflammatory signaling as a key contributor to cancer progression through immune evasion and a pro-tumorigenic microenvironment [19], suggesting these pathways as targets to disrupt tumor–immune crosstalk, an approach with promise in other cancer types [20]. DNA repair mechanisms were similarly dysregulated in metastatic cells, plausibly contributing to mutation accumulation and therapy resistance and supporting DNA repair inhibitors as a candidate sensitizing strategy [21].

The Spearman correlation analysis further underscores this interconnectedness: pathways involved in oxidative stress, immune response, and metabolic regulation showed coordinated variation in activity, consistent with the broader network-pharmacology view that combination therapies targeting multiple co-regulated pathways may outperform single-target approaches [22]. A key contribution of this study is the identification of hub genes—regulators central to both tumor initiation and metastasis and involved in angiogenesis, immune modulation, and cell migration—that may serve as biomarkers or therapeutic targets. This aligns with recent work showing that coordinated hub-gene alterations track with proliferative activity, genomic instability, and disease aggressiveness, supporting the view that ovarian cancer progression reflects dynamic network-level reprogramming rather than isolated gene changes [23]. Because the Spearman correlations were calculated across samples representing distinct biological groups, some of the observed associations may reflect between-group differences rather than within-group co-variation. Therefore, these correlations were interpreted as coordinated pathway activity patterns rather than evidence of direct co-regulation.

The integrated PI3K–AKT model (Figure 4) demonstrates stage-specific rewiring of signaling modules rather than uniform pathway activation: carcinoma was dominated by ECM remodeling and RTK-mediated PI3K activation, ascites by integrin-dependent AKT signaling through EPHA2/ITGB8, and metastasis by inflammatory and survival signaling via *IL6*, *JAK1*, *PTK2*, *AREG*, and NF-κB. This is consistent with Tomuleasa et al., who identified genomic amplification, gain-of-function mutations, and autocrine activation as major mechanisms sustaining constitutive RTK signaling in early tumorigenesis [16], and with Liu et al., who linked altered integrin expression and focal adhesion-mediated survival signaling to anoikis resistance during peritoneal dissemination [17]. Importantly, the present study represents a reanalysis of publicly available datasets that have previously been examined using transcriptomic and hub-gene approaches. GSE36668 has been incorporated into several ovarian cancer studies that identified differentially expressed and hub genes through PPI-based analyses and evaluated their prognostic associations [24,25]. Similarly, GSE73064 has previously been analyzed to identify critical genes and molecular signatures associated with the transition from primary tumor cells to ascites and metastatic cells, including PPI-based hub-gene and survival analyses [26]. Thus, the novelty of the present study does not reside in the generation of new transcriptomic data or in the identification of previously unreported hub genes from these accessions. Rather, we provide a pathway-centered, stage-specific reanalysis that integrates independently derived carcinoma, ascites, and metastasis signatures within a PI3K–AKT-associated network framework. In particular, hub-gene selection based on multiple network-centrality measures was combined with an independent pathway-membership analysis, functional enrichment, exploratory survival analysis, and stage-stratified cross-cohort evaluation in TCGA-OV. This framework allows previously reported expression signals to be interpreted in relation to their stage-specific network context and their consistency across independent patient cohorts, rather than considering individual hub genes in isolation. The resulting model therefore provides an integrative interpretation of how PI3K–AKT-associated molecular programs differ across carcinoma, ascites, and metastatic disease, while recognizing that these associations require experimental validation before mechanistic or causal conclusions can be drawn. Together, our pathway-centered reanalysis extends previous transcriptomic and hub-gene studies by integrating stage-specific PI3K–AKT-associated genes into a unified network-based framework, while further evaluating their functional enrichment, prognostic associations, and cross-cohort reproducibility during HGSOC progression.

Independent, stage-matched validation in TCGA-OV lends further support to this stage-dependent model. The Metastasis-associated hub genes not only replicated but strengthened in Stage IV disease, with *AREG* and *IL6* shifting most notably: *AREG* reversed from a significant protective association in KM Plotter to a strongly significant risk factor in TCGA Stage IV, and IL6 shifted from non-significant to a strongly significant risk factor. This pattern suggests that the prognostic signal of these genes is diluted or obscured when pooled across disease stages and becomes fully apparent only in the biological context from which the genes were originally derived—reinforcing the rationale for stage-specific rather than pan-stage prognostic modeling in HGSOC. The Ascites-associated genes showed concordant, predominantly non-significant associations across both independent cohorts for three of four genes, further supporting their proposed role as transitional, microenvironment-adaptive markers rather than robust prognostic drivers. In contrast, cross-cohort validation of the Carcinoma-associated genes was less straightforward: an initial, near-total reversal of effect direction for most genes was substantially resolved (concordance rising from 8% to 69%) once a cutoff-selection method matched to KM Plotter was applied, indicating that cutoff-method choice—not true biological reversal—explains most of this discordance. This finding echoes a broader methodological caveat: optimal (best) cutoff approaches, while matching common practice in survival-database tools, are prone to overfitting and inflated significance relative to a priori splits such as the median, and cross-cohort comparisons should account for this when interpreting hazard ratio direction. A small number of genes (*FN1*, *SYK*, *IGF1R*, and *TP53*) remained discordant regardless of cutoff method; for *TP53*, this likely reflects the near-universal *TP53* mutation in HGSOC, which decouples transcript abundance from wild-type protein function and is a recognized limitation of expression-based survival analyses for this gene.

Notably, not all hub genes carried a uniformly unfavorable prognosis: *TP53* and *AREG*, despite membership in the proliferative and metastatic signaling modules respectively, were each associated with improved survival. This distinction between network centrality and population-level prognostic value indicates that hub-gene status and survival association are complementary rather than interchangeable. None of the Ascites-associated hub genes reached individual significance, suggesting this transitional module’s prognostic impact may depend on coordinated multi-gene activity; stage-matched cohorts and multi-gene signatures would help clarify this in future work.

Immune pathway dysregulation was another key finding: the tumor microenvironment plays a dual role in cancer, contributing to immune escape and tumor survival [27]. Immune checkpoint inhibitors such as PD-1/PD-L1 blockade could offer a complementary strategy, potentially overcoming chemotherapy resistance and improving immunotherapy efficacy [28]. Angiogenesis’ well-documented role in metastatic spread further supports targeting tumor vasculature as a therapeutic avenue [29]. Finally, EMT—the acquisition of mesenchymal properties and migratory capacity by epithelial cells—was strongly correlated with metastatic progression, with EMT-related gene activation in carcinoma and metastatic stages consistent with its established role in metastasis across cancer types [30]. Together, these findings on inflammation, metabolism, DNA repair, immune modulation, and metastasis point toward stage-specific therapeutic targets; future work should validate them in clinical settings and personalized treatment contexts.

## 4. Materials and Methods

### 4.1. Data Sources and Processing

This study includes two main datasets. The first dataset, GSE36668, includes samples from serous ovarian borderline tumors (GSM898297, GSM898298, GSM898299, GSM898300), serous ovarian carcinoma (GSM898301, GSM898302, GSM898303, GSM898304), and superficial scraping from normal ovaries (GSM898305, GSM898306, GSM898307, GSM898308), which represent the tumor initiation stages [31]. The second dataset, GSE73064, includes samples from primary tumor cells (GSM1881598, GSM1881601, GSM1881604, GSM1881607, GSM1881610), ascites tumor cells (GSM1881599, GSM1881602, GSM1881605, GSM1881608, GSM1881611), and metastasis tumor cells (GSM1881600, GSM1881603, GSM1881606, GSM1881609, GSM1881612), representing tumor progression stages [10]. The data were obtained from the Affymetrix Human Genome U133 plus 2.0 Array platform (Affymetrix, Santa Clara, CA, USA). These data were extracted from CEL files available in the Gene Expression Omnibus (https://www.ncbi.nlm.nih.gov/geo/ (accessed on 18 February 2026)) and were used for bioinformatics analysis in this study.

Raw data were normalized using the RMA (Robust Multi-array Average) method [32]. This method corrects for systematic effects due to experimental processes and improves signal accuracy. After normalization, the data were log2-transformed and prepared for subsequent analysis. Probe IDs were converted to Gene Symbols using the hgu133plus2.db database. Genes with more than one associated probe were summarized using the median of the probe signals for each gene. Given the limited sample size per group (*n* = 4–5), differential expression was assessed using nominal *p*-values (*p* < 0.05) combined with a fold-change threshold (|log2FC| ≥ 2) to balance sensitivity and specificity; this approach, while standard for small microarray cohorts [33], may increase the false-positive rate relative to FDR-adjusted analyses used elsewhere in this study (e.g., GSEA, Spearman correlation).

An important difference between the two datasets should be noted: GSE36668 comprises superficial scrapings of whole ovarian tissue, which necessarily include stromal, vascular, and immune components, whereas GSE73064 comprises EpCAM-microbead-sorted epithelial tumor cells isolated from primary tumors, ascites, and metastases [10]. Because this difference in specimen preparation is aligned with the early-transformation versus tumor-progression axis, cellular composition is a potential confounder for any comparison spanning the two datasets. We therefore quantified stromal and immune content in all samples using ESTIMATE and assessed the sensitivity of the Carcinoma-associated gene set to specimen type (Supplementary Files S5, S7 and S8).

### 4.2. Gene Set Enrichment and Correlation Analysis

To identify differentially enriched biological pathways between the predefined comparison groups, GSEA was performed using Hallmark gene sets from the Molecular Signatures Database (MSigDB). Genes were ranked according to differential expression statistics, and NES were calculated to evaluate pathway enrichment. To evaluate pathway activity at the individual sample level, ssGSEA was performed using the GSVA R package [34]. The resulting ssGSEA enrichment scores were used for downstream pathway activity correlation analysis. Spearman correlation analysis was performed between ssGSEA-derived pathway activity scores. *p*-values were adjusted using the Benjamini–Hochberg method, and correlations with FDR < 0.05 were considered statistically significant.

### 4.3. Network Construction and Hub-Gene Identification

Protein–protein interaction (PPI) networks were constructed and retrieved using the STRING database (https://string-db.org/), accessed on 8 December 2025 [35]. For each pairwise comparison (normal vs. borderline, borderline vs. carcinoma, primary vs. ascites, ascites vs. metastasis), the top 100 highly upregulated differentially expressed genes were used as network input. The analysis results were imported into Cytoscape software (v3.10.1), where additional filters were applied based on degree, betweenness, closeness, and eigenvector centrality to visualize the PPI network [36]. Gephi software (v0.9.2) was used for gene clustering [37]. Hub genes were defined as the top five genes per comparison with the highest composite rank across these four centrality parameters (Figure 3A–F).

Hub genes were defined using two independent selection procedures applied to the same input. First, for each comparison, the top 100 upregulated differentially expressed genes were used as network input, and the five genes with the highest composite rank across four topological parameters (degree, betweenness centrality, closeness centrality, and eigenvector centrality) were designated centrality-based hub genes; these are reported in Figure 3A–F. Second, and independently of centrality, the same top-100 upregulated DEG list for the Carcinoma (Borderline vs. Carcinoma), Ascites (Primary vs. Ascites), and Metastasis (Ascites vs. Metastasis) comparisons was cross-referenced against the PI3K–AKT signaling pathway gene set using Enrichr (https://maayanlab.cloud/Enrichr/ accessed on 8 March 2026) (KEGG_2021_Human and Reactome_2022 libraries) [38], retaining all overlapping genes. This yielded 13 Carcinoma-, 4 Ascites-, and 7 Metastasis-associated genes, which form the basis of Figure 3H, Figure 4 and Figure 5. Because these two procedures apply different selection criteria (network centrality versus pathway membership) to the same DEG pool, the resulting gene sets are distinct and non-nested, and are not expected to overlap.

### 4.4. Functional and Survival Validation

Upregulated hub genes from the Carcinoma, Ascites, and Metastasis groups were independently mapped to the 10 components of the 2011 Hallmarks of Cancer framework—eight hallmarks (sustaining proliferative signaling, evading growth suppressors, resisting cell death, enabling replicative immortality, inducing or accessing the vasculature, activating invasion and metastasis, reprogramming cellular metabolism, and avoiding immune destruction) and two enabling characteristics (genome instability and mutation, and tumor-promoting inflammation), using the https://cancerhallmarks.com/ platform [39,40], the core cancer hallmark gene set (*n* = 1574 genes) was used as the reference hallmark annotation database for overrepresentation analysis, accessed on 12 December 2025. Because genes prioritized through PPI-based network analysis are typically highly connected and extensively characterized, enrichment against cancer-specific hallmark annotations may partially reflect existing biological knowledge and annotation density. Therefore, Hallmark enrichment was considered as a contextual functional characterization of network-prioritized genes rather than an independent discovery of cancer-associated pathways. The background universe for Fisher’s exact test consisted of all genes retained after probe annotation and filtering from the Affymetrix Human Genome U133 Plus 2.0 Array platform. This background was selected to represent the set of genes detectable within the analyzed transcriptomic datasets and to reduce bias introduced by comparing hub genes only against a cancer-focused annotation set.

To assess the prognostic relevance of PI3K–AKT-associated hub genes identified in the Carcinoma, Ascites, and Metastasis groups, overall survival (OS) analysis was performed for each gene individually using the KM Plotter ovarian cancer database (https://kmplot.com/analysis/) [41], accessed on 12 December 2025. Samples were dichotomized using the auto-select best cutoff (percentile-based optimal threshold) method, and Kaplan–Meier curves were generated with hazard ratios (HR), 95% confidence intervals, and log-rank *p*-values calculated for each representative probe using the user-selected probe set option, with biased arrays excluded from the analysis. Survival differences between high- and low-expression groups were assessed using the log-rank test, with hazard ratios and 95% confidence intervals estimated via Cox proportional hazards regression, both implemented within the KM Plotter platform. As *p*-values generated by KM Plotter are not corrected for multiple hypothesis testing by default, survival results were interpreted as exploratory/hypothesis-generating rather than confirmatory. HR, 95% CI, and log-rank *p*-values obtained from individual KM Plotter outputs were subsequently compiled and visualized as a single integrated forest plot using the ggplot2 package in R, stratified by disease stage (Carcinoma, Ascites, and Metastasis). Because the KM Plotter cohort predominantly comprises primary tumor specimens collected at diagnosis, these results reflect the prognostic relevance of hub-gene expression in primary disease rather than direct functional validation within the ascites or metastatic microenvironments.

### 4.5. Statistical Analysis and Software

All analyses were performed using R version 4.5.2 (R Foundation for Statistical Computing, Vienna, Austria). Differentially expressed genes (DEGs) were identified using the limma package (version 3.64.3), and Benjamini–Hochberg FDR correction (threshold < 0.05) was applied to correlation and GSEA results as described above. ssGSEA scores were computed using the GSVA package (version 2.2.1), while msigdbr (version 26.1.1) was used for gene set retrieval. dplyr (version 1.2.1) and tidyr (version 1.3.2) were used for data handling, and ggplot2 (version 4.0.3) was used for visualizations, including bubble plots and the integrated forest plot of survival hazard ratios. Schematic diagrams, including the two-stage tumor progression model and the integrated PI3K–AKT pathway schematic, were created using Whimsical (www.whimsical.com).

### 4.6. TCGA-OV Stage-Specific Cross-Cohort Validation

Because the KM Plotter cohort pools tumors across all disease stages and lacks metadata separating ascitic or distant-metastatic specimens from primary tumors, an independent, stage-stratified validation was performed using TCGA-OV (accessed via curatedTCGAData, version 1.30.0) to more directly test the biological context each hub-gene subset was originally derived from. Carcinoma-associated hub genes were evaluated in non-metastatic, Stage I-III TCGA-OV samples (*n* = 358), matching the early-transformation origin of the GSE36668-derived Carcinoma group. Because TCGA-OV consists almost exclusively of surgically resected primary tumor tissue and lacks ascitic fluid specimens, both Ascites- and Metastasis-associated hub genes were evaluated in Stage IV samples, using AJCC Stage IV classification as a proxy for advanced, disseminated disease; this is a necessary approximation rather than a direct biological match to the ascites- or metastasis-derived origin of these gene sets (see Section 5, Limitations). Univariate Cox regression was performed for each gene using expression dichotomized at the sample median. For the Carcinoma group, a sensitivity analysis was additionally performed using an optimal (best) cutoff, identified via survminer (version 0.5.2) using surv_cutpoint(), to match the cutoff-selection strategy used by KM Plotter (auto-select best cutoff) and to test whether cutoff method, rather than biological reversal, explained cross-cohort discordance. Cross-cohort concordance was defined as directional agreement (hazard ratio above or below 1) between the TCGA-OV and KM Plotter estimates for each gene, summarized using Spearman correlation of log-transformed hazard ratios and the proportion of directionally concordant genes overall and by hub-gene subgroup.

## 5. Limitations

This study has several limitations. First, the small sample size per group (*n* = 4–5) limits statistical power and may increase susceptibility to false-positive calls despite established normalization and fold-change thresholds; relatedly, the Carcinoma group derives from a distinct dataset (GSE36668) than Ascites and Metastasis (GSE73064), so the integrated PI3K–AKT network (Figure 3H) and schematic model (Figure 4) represent a conceptual, cross-dataset trajectory rather than a batch-corrected comparison. Second, hub-gene identification relied on network topology rather than orthogonal experimental validation (e.g., qPCR, immunohistochemistry, functional knockdown). Third, survival validation via KM Plotter reflects primary tumor specimens at diagnosis and does not directly capture ascitic or metastatic prognostic associations; we partly addressed this through independent, stage-matched TCGA-OV validation (Carcinoma: Stage I–III; Ascites/Metastasis: Stage IV), but TCGA-OV consists almost exclusively of resected primary tumor tissue and lacks literal ascitic fluid specimens, so the TCGA “Ascites” comparison should be understood as a Stage IV proxy for disseminated disease rather than a direct biological match; the TCGA Ascites subgroup was also small, limiting statistical power. Relatedly, the Carcinoma cross-cohort comparison showed substantial sensitivity to cutoff-selection method (23% directional concordance with median split versus 69% with a KM Plotter-matched optimal cutoff), underscoring that optimal-cutoff hazard ratios—though useful for methodological consistency across cohorts—are more prone to instability and overfitting than a priori splits and should be interpreted cautiously, particularly for genes with modest effect sizes. Finally, as a microarray-based study, this work cannot distinguish mutational status (e.g., *TP53* gain- versus loss-of-function) from transcript abundance; this is consistent with *TP53* being one of the few genes whose survival association direction remained discordant between KM Plotter and TCGA-OV even after cutoff-method matching. Larger prospective cohorts, orthogonal validation, and mutation-resolved sequencing are warranted to extend these findings. Another limitation is the potential annotation bias associated with cancer hallmark enrichment analysis. Since the analyzed genes were prioritized through PPI network topology, they are likely enriched for highly connected and well-studied cancer-related genes. Consequently, overlap with established cancer hallmark gene sets may partly reflect prior biological annotation rather than exclusively representing newly identified cancer-specific processes. These findings should therefore be interpreted as functional contextualization of prioritized network modules rather than definitive evidence of pathway discovery. An additional limitation concerns cellular composition. The two source datasets differ in specimen preparation (whole-tissue scrapings in GSE36668 versus EpCAM-sorted epithelial cells in GSE73064), and this difference is aligned with the stage axis. Direct cross-dataset comparison of the 13 Carcinoma-associated PI3K–AKT genes between GSE36668-Carcinoma and GSE73064-Primary, both of which represent early-stage/primary disease, showed that three genes (*FN1*, *CCND1*, *SYK*) differed by less than 0.1 log2 units, four genes (*TP53*, *COL6A2*, *IGF1R*, *CSF1R*) showed moderate differences (0.4–1.0 log2 units), and six ECM/collagen genes (*COL1A1*, *PDGFRA*, *LAMB1*, *THBS2*, *COL1A2*, *COL6A3*) were substantially higher (1.4–2.8 log2 units) in whole-tissue samples (Supplementary File S8). The Carcinoma signature therefore represents a mixed tumor-stroma signal, and only *FN1*, *CCND1*, and *SYK* can be regarded as having strong evidence of tumor-cell-intrinsic dysregulation on the basis of these data. Within-dataset correlation of individual hub genes with ESTIMATE StromalScore was uninformative at this sample size, with no correlation surviving FDR correction across the 24 genes tested (Supplementary File S8). Purity-adjusted analysis in larger, uniformly prepared cohorts would be required to resolve the tumor-intrinsic component of this signature definitively.

## 6. Conclusions

This study provides a comprehensive systems-level characterization of molecular alterations associated with the progression of high-grade serous ovarian cancer (HGSOC) from early tumorigenesis to metastatic disease. Integrating differential gene expression, network topology, pathway enrichment, and correlation analyses revealed a progressive shift from epithelial homeostasis toward inflammatory signaling, metabolic reprogramming, extracellular matrix remodeling, and immune adaptation during disease progression. Hub-gene and protein–protein interaction analyses identified stage-specific molecular regulators, while Reactome pathway reconstruction highlighted a transition from proliferative signaling in early-stage tumors to survival- and microenvironment-associated programs in advanced disease. Finally, the integrated PI3K–AKT signaling model provided a network-based framework for linking these molecular alterations into a coherent stage-dependent signaling network, emphasizing the dynamic interplay between RTK, integrin, PI3K–AKT, MAPK, and NF-κB pathways. Collectively, these findings improve our understanding of HGSOC progression and identify stage-specific molecular candidates that may serve as biomarkers or therapeutic targets for future experimental and clinical investigations.

## Figures and Tables

**Table 1:** Stage-specific hub genes identified across the ovarian cancer progression sequence.

Stage	Dataset	n	Hub Gene
Normal	GSE36668	4	*HSP90AB1*, *PIK3R1*, *FGF2*, *CDC42*, *THBS1*
Borderline	GSE36668	4	*FN1*, *CDH1*, *CCND1*, *EPCAM*, *MUC1*
Carcinoma	GSE36668	4	*BCL2*, *SMAD4*, *CCDC114*, *CFAP52*, *FOXO1*
Primary	GSE73064	5	*CDKN2A*, *AGO2*, *SRSF1*, *SMAD3*, *AGO3*
Ascites	GSE73064	5	*PTPRC*, *ITGB2*, *TYROBP*, *CD4*, *ITGAM*
Metastasis	GSE73064	5	*TYROBP*, *ITGB2*, *ITGAM*, *PTPRC*, *FCER1G*

## Data Availability

The datasets produced and/or analyzed in this study are accessible from the GSE repository at [GSE73064] (https://www.ncbi.nlm.nih.gov/geo/query/acc.cgi?acc=GSE73064) and [GSE36668] (https://www.ncbi.nlm.nih.gov/geo/query/acc.cgi?acc=GSE36668), both accessed on 18 February 2026.

## Supplementary Materials

The following supporting information can be downloaded at: https://www.mdpi.com/article/10.3390/ijms27198700/s1.
