# Supplementary material for: Stage-Specific Rewiring of PI3K–AKT Signaling During Serous Ovarian Cancer Progression: An Integrated Transcriptomic and Network Analysis
Source: Int J Mol Sci. 2026 Sep 29;27(19):8700. doi: 10.3390/ijms27198700 (PMC13665355; doi:10.3390/ijms27198700)
Supplement: Supplementary file 1 [file ijms-27-08700-s001.zip › Supplementary Tables.pdf]

Supplementary Table S1. Significantly enriched GO terms (BP, CC, MF) for PI3K-AKT-associated hub genes in Carcinoma, Ascites, and Metastasis (Enrichr; adjusted $p < 0.05$).

GO	Carcinoma	Ascites	Metastasis
**BP**	Peptidyl-tyrosine Phosphorylation	Epidermis Development	Positive Regulation of Fibroblast Migration
**BP**	Positive Regulation of Cell Migration	Regulation of Angiogenesis	Negative Regulation of Cell Adhesion Mediated by Integrin
**BP**	Positive Regulation of Cell Motility	Positive Regulation of Cell Motility	Interleukin-6-mediated Signaling Pathway
**BP**	Positive Regulation of Cellular Process	Positive Regulation of Cell Migration	Positive Regulation of Macrophage Chemotaxis
**BP**	Odontogenesis	Regulation of Cell Migration	Positive Regulation of Homotypic Cell-Cell Adhesion
**CC**	Endoplasmic Reticulum Lumen	Focal Adhesion	RISC Complex
**CC**	Supramolecular Fiber	Cell-substrate Junction	Endoplasmic Reticulum Lumen
**CC**	Intracellular Organelle Lumen	Basement Membrane	Endocytic Vesicle Lumen
**CC**	Microfibril	Tight Junction	Focal Adhesion
**CC**	Protein Kinase Complex	Extracellular Exosome	Cell-substrate Junction
**MF**	Platelet-derived Growth Factor Binding	Ephrin Receptor Activity	Interleukin-6 Receptor Binding
**MF**	Vascular Endothelial Growth Factor Receptor Activity	Transmembrane Receptor Protein Kinase Activity	JUN Kinase Binding
**MF**	Insulin-like Growth Factor I Binding	Protein Tyrosine Kinase Activity	Protein Tyrosine Kinase Activity
**MF**	Protein Tyrosine Kinase Activity	Cadherin Binding	Phosphatase Binding
**MF**	Protease Binding	Transmembrane Receptor Protein Tyrosine Kinase Activity	Ephrin Receptor Activity

Supplementary Table S2. Cross-cohort validation of PI3K-AKT pathway hub genes: KM Plotter vs. TCGA-OV hazard ratios (95% CI available in source data), stratified by AJCC stage (Carcinoma: Stage I-III, n=358; Ascites/Metastasis: Stage IV). Carcinoma TCGA estimates use the KM Plotter-matched optimal-cutoff method (see Supplementary Figure S4 for the median-split comparison). Overall directional concordance: 17/24 genes (71%).

Gene	Group	HR (KM Plotter)	P (KM Plotter)	HR (TCGA-OV)	P (TCGA-OV)	Concordant
CCND1	Carcinoma	1.08	0.24	1.42	0.0287	Yes
COL1A1	Carcinoma	1.55	1.8e-10	1.34	0.0446	Yes
COL1A2	Carcinoma	1.28	0.0005	1.27	0.1441	Yes
COL6A2	Carcinoma	1.37	6.3e-06	1.19	0.233	Yes
COL6A3	Carcinoma	1.18	0.011	1.37	0.0502	Yes
CSF1R	Carcinoma	1.20	0.0074	1.64	0.0012	Yes
FN1	Carcinoma	1.42	7.3e-07	0.77	0.1251	No
IGF1R	Carcinoma	0.84	0.064	1.21	0.2331	No
LAMB1	Carcinoma	1.23	0.0048	1.39	0.0369	Yes
PDGFRA	Carcinoma	1.34	9.5e-05	1.49	0.0188	Yes
SYK	Carcinoma	1.18	0.087	0.71	0.0259	No
THBS2	Carcinoma	1.54	4.3e-10	1.40	0.0312	Yes
TP53	Carcinoma	0.76	5.3e-05	1.37	0.0438	No
EPHA2	Ascites	0.91	0.17	0.99	0.9745	Yes
ITGB8	Ascites	1.12	0.24	1.78	0.1951	Yes
LAMB3	Ascites	1.05	0.42	0.50	0.1185	No
LAMC2	Ascites	0.89	0.067	0.98	0.9524	Yes
AREG	Metastasis	0.87	0.03	1.25	1.59e-08	No
EIF4E	Metastasis	1.24	0.0029	2.24	0.0001	Yes
HSP90B1	Metastasis	1.09	0.19	3.26	7.23e-07	Yes
IL6	Metastasis	0.91	0.16	1.35	1.04e-07	No
JAK1	Metastasis	1.26	0.0014	3.04	0.0075	Yes
PTK2	Metastasis	1.25	0.0011	2.47	4.63e-06	Yes
THBS1	Metastasis	1.39	2.8e-07	1.63	6.64e-05	Yes
